# Enhanced power factor via the control of structural phase transition in SnSe

**DOI:** 10.1038/srep26193

**Published:** 2016-05-19

**Authors:** Hulei Yu, Shuai Dai, Yue Chen

**Affiliations:** 1 Department of Mechanical Engineering, The University of Hong Kong, Pokfulam Road, Hong Kong SAR, China

## Abstract

Tin selenide has attracted much research interest due to its unprecedentedly high thermoelectric figure of merit (ZT). For real applications, it is desirable to increase the ZT value in the lower-temperature range, as the peak ZT value currently exists near the melting point. It is shown in this paper that the structural phase transition plays an important role in boosting the ZT value of SnSe in the lower-temperature range, as the Cmcm phase is found to have a much higher power factor than the Pnma phase. Furthermore, hydrostatic pressure is predicted to be extremely effective in tuning the phase transition temperature based on ab-initio molecular dynamic simulations; a remarkable decrease in the phase transition temperature is found when a hydrostatic pressure is applied. Dynamical stabilities are investigated based on phonon calculations, providing deeper insight into the pressure effects. Accurate band structures are obtained using the modified Becke-Johnson correction, allowing reliable prediction of the electrical transport properties. The effects of hydrostatic pressure on the thermal transport properties are also discussed. Hydrostatic pressure is shown to be efficient in manipulating the transport properties via the control of phase transition temperature in SnSe, paving a new path for enhancing its thermoelectric efficiency.

As promising candidates for renewable energy, thermoelectric (TE) materials are widely investigated for the reversible conversion between electricity and heat^1^,^2^,^3^. The thermoelectric efficiency is determined by the figure of merit:


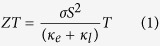


where Seebeck coefficient (*S*), electrical conductivity (*σ*), electronic and lattice thermal conductivities (*κ*_*e*_ and *κ*_*l*_) are to be simultaneously optimized to obtain a high power factor (*PF* = *σS*^2^) and a low thermal conductivity (*κ* = *κ*_*e*_ + *κ*_*l*_). It was recently reported that SnSe, a narrow band gap semiconductor, exhibits an unprecedentedly high *ZT* value of about 2.6 ± 0.3 at 923 *K*^4^, which makes SnSe one of the most promising TE materials. As SnSe only contains earth abundant elements that are relatively low toxic comparing to lead or tellurium, it is extremely attractive for potential large-scale applications^5^,^6^,^7^. It has been shown that the *ZT* value of SnSe increases dramatically at around 800 *K* and reaches a maximum at 923 *K*^4^. Nonetheless, the *ZT* value is rather low over a large temperature range under 800 *K*, making it inefficient in practical applications. Significant progress has recently been made by Zhao *et al*.^8^, who discovered that the *ZT* value of SnSe in the low temperature range can be dramatically increased by hole doping. In this paper, we propose an alternative strategy for enhancing SnSe’s energy conversion efficiency by manipulating its structural phase transitional temperature through pressure.

SnSe adopts a layered orthorhombic crystal structure (*Pnma*), which can be derived from a distortion of the rock-salt structure, at ambient temperature^9^, and undergoes a continuous phase transition to the *Cmcm* structure between 750 *K* ~ 800 *K*^4^,^10^. The unit cells of both structures contain eight atoms that are covalently bonded to three neighbors, as illustrated in Fig. 1. Double atomic layers stack along the *a* axis, where the Sn-Se bonding is relatively weaker, and corrugated layers resulting from the distortion can be observed from the *b* axis. Based on the neutron diffraction method, Chattopadhyay *et al*. investigated the structural phase transition in SnSe and found that the crystal structure changed from the *Pnma* (a = 11.49 Å, b = 4.44 Å, c = 4.135 Å) to the *Cmcm* (a = 4.31 Å, b = 11.70 Å, c = 4.31 Å) space group at 807 *K*^11^. Chattopadhyay *et al*. also studied the structure of SnSe under pressure by energy-dispersive x-ray diffraction, while no evidence of phase transition was found^12^. On the other hand, more recent study using angle-dispersive synchrotron x-ray powder diffraction revealed a hydrostatic pressure-induced phase transition at 10.5 *GPa*^13^. Based on density functional theory (DFT), Alptekin *et al*.^14^ reproduced the hydrostatic pressure-induced phase transition, and further predicted that a much lower uniaxial stress (3 *GPa*) was required to induce the structural phase transition. However, lattice dynamics are yet to be investigated to provide further evidence to support this prediction.

Since the structural phase transition in SnSe may significantly affect the TE performance and it can be induced by both temperature and pressure, it is of great significance to have a deeper understanding of the relationship between the transport properties and the phase transition. Therefore, we have studied the energetics and lattice dynamics in SnSe under both hydrostatic pressure and uniaxial stress using DFT and ab-initio molecular dynamics (AIMD) simulations. The structural phase transition from *Pnma* to *Cmcm* is directly observed in our AIMD simulation, and a strong dependence of the phase transition temperature on pressure is identified. Based on the phonon dispersion and total energy calculations, it is found that the high-temperature *Cmcm* phase tends to exhibit better stability as the applied hydrostatic pressure increases. On the other hand, phonon calculations indicate a dynamical instability of the *Cmcm* structure under uniaxial stress. The structural phase transition temperature is found to be significantly lowered when a relatively low hydrostatic pressure is applied, which provides a potential route to manipulate the thermoelectric performance of SnSe. The effects of hydrostatic pressure on the electrical transport properties are also investigated by combing DFT and Boltzmann transport theory. It is found that the band gap of SnSe decreases linearly as pressure increases, while the electrical transport properties of the two competing phases are less influenced by external pressure. Completing the discussion on the *ZT* value, the thermal transport properties are also investigated.

## Computational details

DFT calculations are performed using the Vienna ab-initio simulation package (VASP)^15^. The projector-augmented wave method (PAW)^16^ combined with the generalized gradient approximation (GGA) of Perdew-Burke and Ernzerhof (PBE)^17^ is applied. Forces and the full stress tensor are calculated and used to relax crystal structures into their ground states. The Brillouin zones are meshed using the gamma-centered Monkhorst-Pack method^18^ with a density of about 2*π* × 0.03 Å^−1^. A plane-wave energy cut-off of 350 *eV* is specified and the convergence criteria for electronic self-consistence calculations is set to be 10^−6 ^*eV* to ensure a sufficient accuracy. The unit cells^11^,^19^ of the *Pnma* and *Cmcm* structures are fully optimized under different hydrostatic pressures and uniaxial stresses. For phonon calculations, a 2 × 4 × 4 supercell containing 256 atoms is adopted to study the phonon and *Grüneisen* dispersions based on the small displacement method^20^. To ensure accurate calculations of atomic forces, we increase the total energy convergence criteria to 10^−8 ^*eV*. Volume changes of ±1% are used in *Grüneisen* dispersion calculations. The supercell containing 256 atoms is also used in AIMD simulations, in which atomic forces are calculated directly from the electronic structure based on DFT. The system is coupled to a Nose thermostat to impose an NVT ensemble. A time step of 5 *fs* is applied, and the system is initially equilibrated for 1000 MD steps, while data is only collected from the subsequent 10000 steps.

It is well known that electronic band gap is usually underestimated in GGA, resulting in unreliable predictions of electrical transport properties. For more accurate results, we have applied the modified Becke-Johnson method^21^ for band structure calculations. The electrical transport properties of SnSe under different pressures are calculated based on the Boltzmann transport theory^22^,^23^ as implemented in the BoltzTraP code^24^. For accurate predictions of the transport properties, electronic band structures are calculated on a denser mesh with around 15000 k-points in the Brillouin zone. The lower limit of the lattice thermal conductivity is calculated using the following equation^25^:


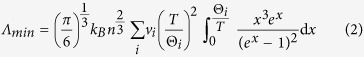


where *v*, Θ and *n* are phonon group velocity, Debye temperature and the number density of atoms, respectively.

## Results and Discussions

### Energetics and lattice dynamics at 0 *K* under hydrostatic pressure

To study the pressure-induced phase transition under hydrostatic pressure, we have compared the Gibbs free energy (*G*) of the *Pnma* and *Cmcm* phases. In the case of *T* = 0 *K, G* is simply equal to the enthalpy (*H*):


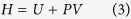


Where *U* is the internal energy, *P* is the external pressure and *V* is the equilibrium volume. The enthalpies of the two competing structures (*Pnma* and *Cmcm*) are calculated after fully relaxations of the unit cells under a range of hydrostatic pressures from 0 *GPa* to 30 *GPa*. The enthalpy differences between the two phases are compared at different hydrostatic pressures (Fig. 2(a)). It is seen that *Pnma* has lower enthalpies below 8 *GPa*, which indicates *Pnma* is energetically preferable at low pressures. The enthalpy difference decreases with increasing pressure and becomes zero when *P* > 8 *GPa*, in agreement with previous studies^14^,^13^.

The enthalpy differences under hydrostatic pressure can be better understood by further analyzing the geometric parameters of the two competing crystal structures as shown in the supplementary materials. It is found that the lattice constants and atomic coordinates of the *Pnma* and *Cmcm* structures become identical at pressure over about 12 *GPa*, providing further evidences of a continuous phase transition from *Pnma* to *Cmcm* with increasing hydrostatic pressure. Our calculations are in good agreement with recent experimental observations of a critical pressure of 10.5(3)*GPa* for inducing the phase transition^13^.

Although the *Cmcm* phase is energetically preferable under hydrostatic pressure at *T* = 0 *K*, its dynamical stability has not yet been investigated. We have thus calculated the phonon dispersions of the *Cmcm* phase under hydrostatic pressures of 0 *GPa*, 4 *GPa* and 10 *GPa* (see Fig. 2(b–d)). It is found that the *Cmcm* phase has large imaginary phonon frequencies near the Γ point at 0 *GPa*. These imaginary frequencies indicate that the *Cmcm* phase is dynamically unstable at ambient pressure. As the hydrostatic pressure increases, the imaginary phonon frequency is found to be gradually reduced and it completely disappears at 10 *GPa*, indicating that the *Cmcm* phase is dynamically stabilized. It is worth noting that the phonon calculations are based on harmonic approximation, i.e., phonon interactions are neglected. Therefore, the phonon dispersions only describe the lattice dynamics at *T* = 0 *K*. As the *Cmcm* phase is observed at high temperature, it is anticipated that the imaginary phonon frequencies at 0 *GPa* will disappear at elevated temperatures due to lattice anharmonicity.

### Hydrostatic pressure effects at elevated temperatures

**Figure 1:**
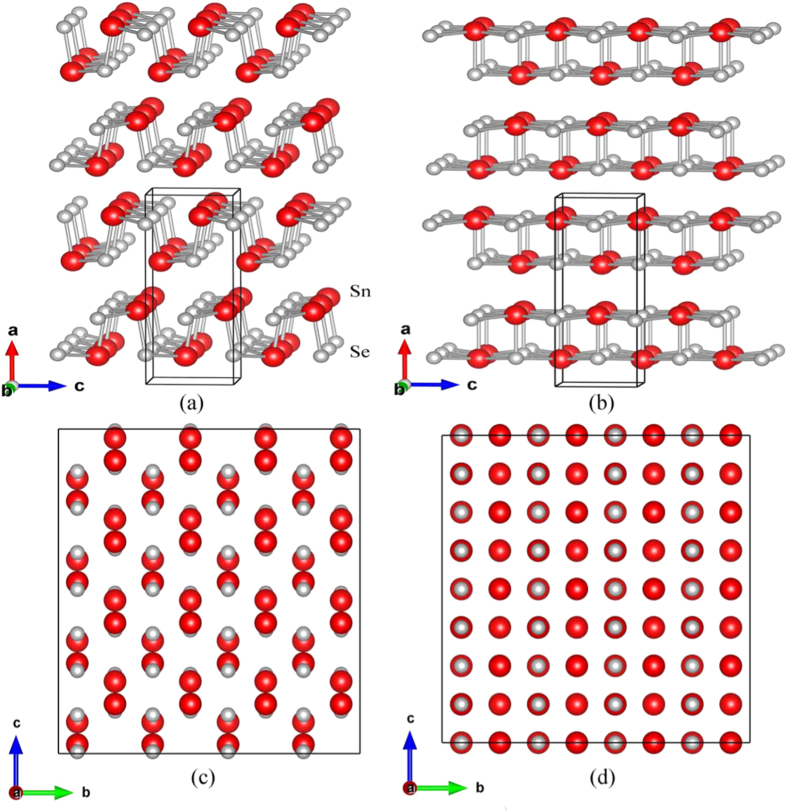
Side views of the *Pnma* (**a**) and *Cmcm* (**b**) crystal structures of SnSe. Top views of the *Pnma* and *Cmcm* structures are shown in panel (**c**,**d**), respectively.

**Figure 2:**
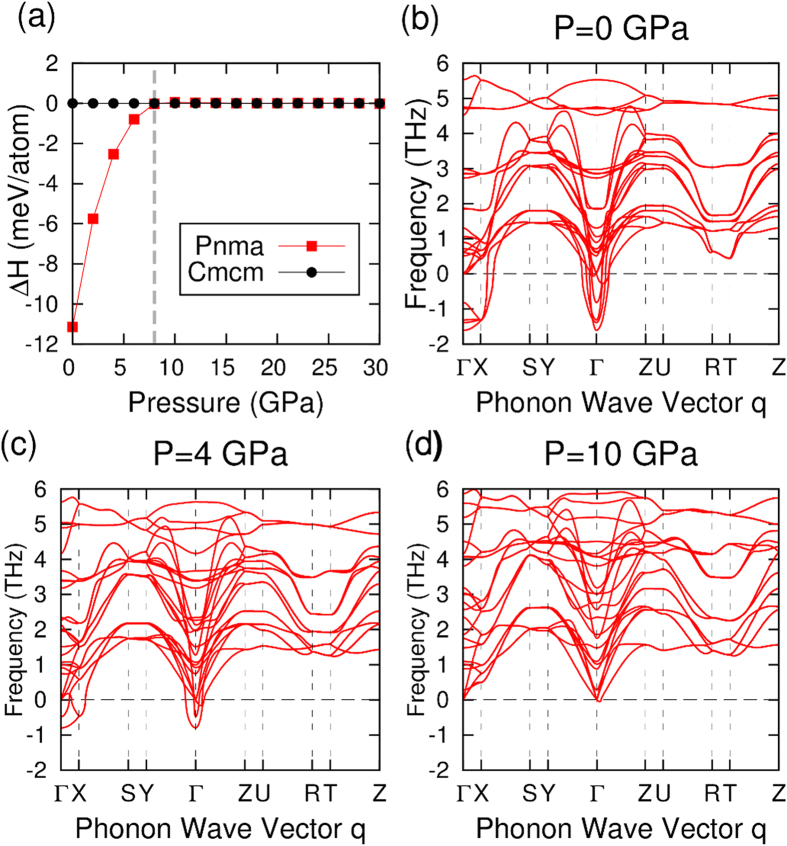
The theoretical enthalpy (**a**) of the *Pnma* versus the *Cmcm* phase as a function of hydrostatic pressure from 0 *GPa* to 30 *GPa*. The gray dashed line is located at *P* = 8 *GPa*. Phonon dispersions of the *Cmcm* phase under hydrostatic pressure of 0 *GPa* (**b**), 4 *GPa* (**c**) and 10 *GPa* (**d**).

**Figure 3:**
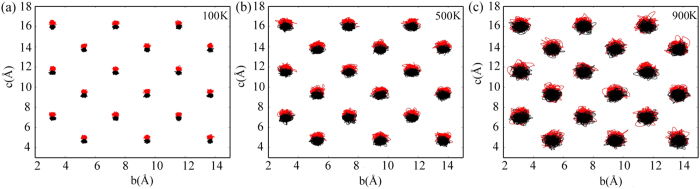
Trajectory projections of the Sn (red) and Se (black) atoms on the *bc* plane for AIMD simulations at (**a**) 100 *K*, (**b**) 500 *K* and (**c**) 900 *K*.

**Figure 4:**
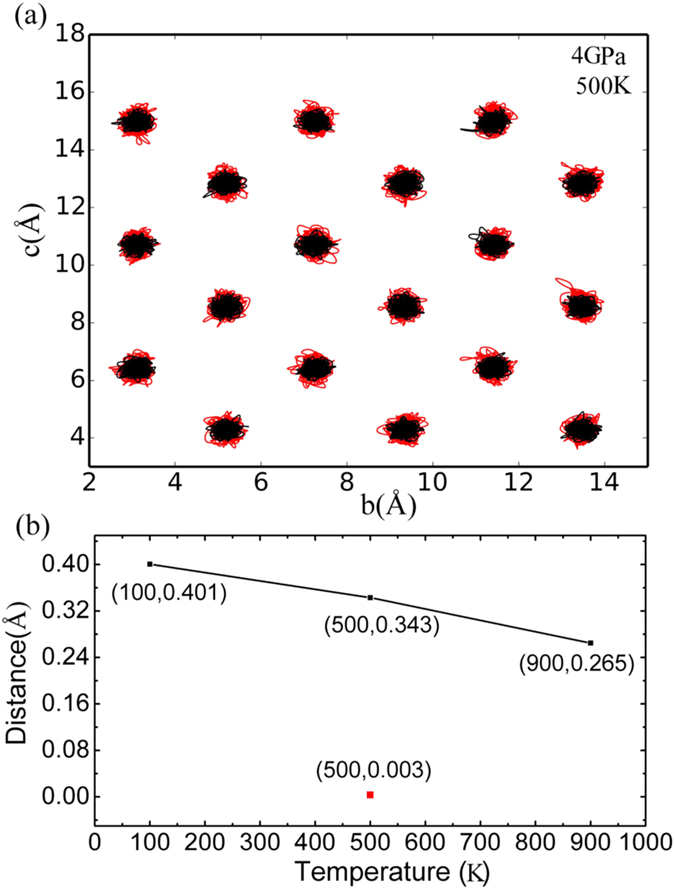
(**a**) Trajectory projections of the Sn (red) and Se (black) atoms on the *bc* plane during AIMD simulations at T = 500 *K* under a hydrostatic pressure of 4 *GPa*. (**b**) Atomic distances between the adjacent Sn and Se atoms along the *c* axis; the solid line represents the atomic distances at ambient pressure and the discrete red square represents the atomic distance at 500 *K* under a hydrostatic pressure of 4 *GPa*.

Although the *Cmcm* phase can be stabilized at *T* = 0 *K* under a hydrostatic pressure greater than 10 *GPa*, it is of practical significance if the required pressure for the phase transition can be further reduced. By comparing the crystal structures of the two competing phases, an obvious difference on the projection of the atomic positions onto the *bc* plane can be seen (Fig. 1). This difference is used to identify the structural phase transition during AIMD simulations. We have performed AIMD at different temperatures and pressures, and the trajectories of the Sn and Se atoms in one double-layer structure are projected onto the *bc* plane, as shown in Figs 3 and 4(a). Atomic distances between the adjacent Sn and Se atoms along the *c* axis are calculated and summarized in Fig. 4(b). As expected, the stable structure at low temperature is *Pnma*, in which Sn and Se do not align on top of each other along the *a* axis. It is seen that the average positions of the Sn and Se atoms tend to align along the *a* axis with increasing temperature, resulting in a continuous phase transition from *Pnma* to *Cmcm*. It is worth noting that the phase transition has not completed even at 900 *K* in our simulations, which overestimate the experimental value of 825 *K*^12^; the overestimation may be attributed to the constraints of invariant unit cell in the NVT ensemble, while *Pnma* and *Cmcm* have minor differences in the lattice parameters.

In addition, we have also performed AIMD simulations at 500 *K* using the relaxed supercell of *Pnma* under a hydrostatic pressure of 4 *GPa*. It is seen from Fig. 4(a) that the phase transition from *Pnma* to *Cmcm* completes at 500 *K* when a hydrostatic pressure of 4 *GPa* is applied; this is in contrast to the simulation at 500 *K* without external pressure (Fig. 3(b)), in which the phase transition has not completed. In other words, hydrostatic pressure promotes the transformation from *Pnma* to *Cmcm* and the phase transition temperature may be effectively decreased via pressure.

**Figure 5:**
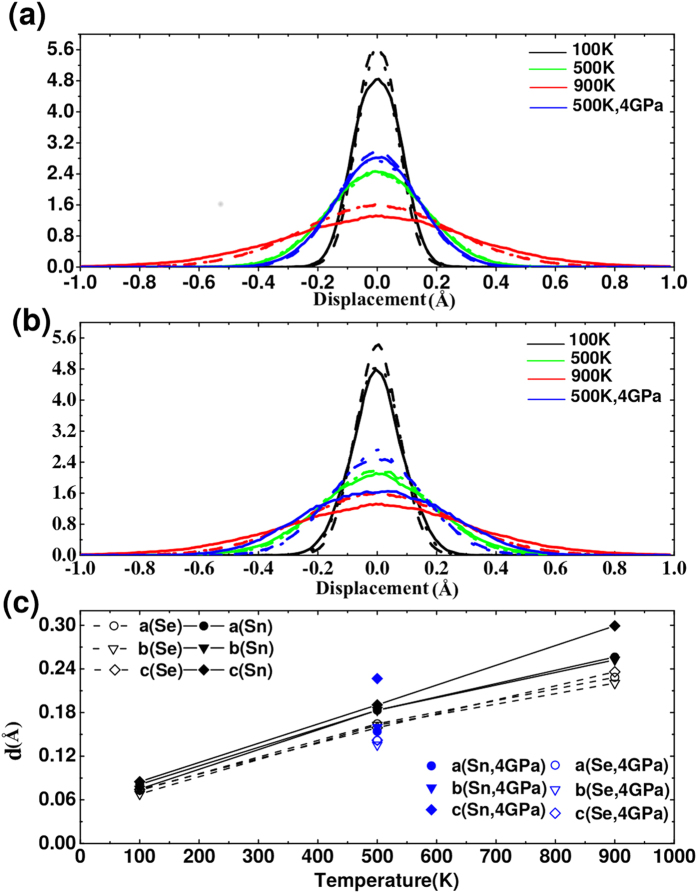
Probability distributions of the Se (**a**) and Sn (**b**) atoms; the dotted, dashed and solid lines represent the atomic vibrations along the *a, b* and *c* axes, respectively. (**c**) Root mean square of the atomic vibration at different temperatures along the *a, b* and *c* axes.

Based on our AIMD simulations, we have also investigated the probability distributions of the Sn and Se atoms as well as their vibrational amplitudes. As can be seen from Fig. 5, the probability distributions exhibit a symmetric behavior with the maximum values sitting in the original equilibrium atomic positions. The probability distributions gradually broaden with increasing temperature, indicating an increase in the atomic vibrational amplitudes. By calculating the root mean square of atomic displacements, as shown in Fig. 5(c), both Sn and Se atoms have similar vibrational amplitudes along different directions at low temperature. On the other hand, it is interesting that the Sn atoms have a larger vibrational amplitude along the *c* axis at 900 *K*, at which the system represents approximately the *Cmcm* structure. Similar behavior is also evidenced when a hydrostatic pressure of 4 *GPa* is applied to the system; the atomic vibrational amplitudes along the *a* and *b* axes decrease as expected comparing to their values under ambient pressure at the same temperature. Surprisingly, the atomic vibrational amplitude of the Sn atoms along the *c* axis increases dramatically, indicating weaker bonding along this direction in the *Cmcm* phase. In contrast, the Se atoms show similar vibrational amplitudes along different directions. The strong anisotropy in the transport properties of SnSe is thus believed to be related to the anomalous vibrational behavior of the Sn atoms.

### Effects of uniaxial stress

In addition to the hydrostatic pressure, we have also investigated the effects of uniaxial stress on the structural phase transition. Uniaxial stresses along all three axes of the unit cells have been considered, while the *Pnma* to *Cmcm* transition is only observed when the *c* axis is compressed. The changes in the lattice parameters and atomic coordinates regarding to the applied uniaxial stress along the *c* axis are included in the supplementary materials. It is found in the structural relaxations that *Pnma* transforms to *Cmcm* when the uniaxial stress along the *c* axis is greater than 3.5 *GPa*, in agreement with previous theoretical predictions^14^.

**Figure 6:**
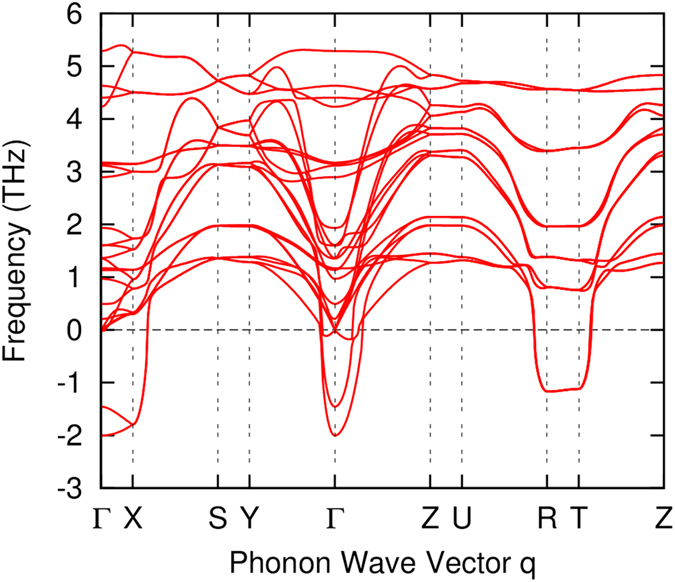
Phonon dispersion of the *Cmcm* phase under a uniaxial compression of about 4 *GPa* along the *c* axis. Large soft phonon modes with imaginary frequencies (shown as negative values) are observed.

It is worth noting that although the *Cmcm* phase becomes energetically preferable under uniaxial compression, its dynamical stability needs to be further studied to confirm the structural phase transition. Phonon dispersion of the *Cmcm* phase under a uniaxial stress of about 4 *GPa* along the *c* axis has been computed, as shown in Fig. 6. It is seen that there exists large imaginary phonon frequencies, indicating that the *Cmcm* phase is dynamically unstable. Phonon calculations under higher uniaxial compression have also been performed, while no evidence is found on the hardening of the soft phonon modes. Therefore, the structural phase transition under uniaxial compression is relatively more complicated, as new crystal structures related to the soft phonon modes may exist. In the following discussions on the transport properties, we will only focus on the effects of hydrostatic pressure.

### Pressure effects on electronic band structures

The band gap of the *Pnma* phase under ambient pressure has been calculated using both GGA and LDA, and the results are summarized in Table 1. It is well known that conventional DFT calculations usually underestimate electronic band gaps. As expected, the experimentally measured band gap of the *Pnma* phase, which is about 0.86 *eV*^4^, is larger than the values obtained from GGA and LDA. To obtain more accurate band gaps from theory, more sophisticated GW method^26^ or the mBJ method^27^,^28^,^29^ may be applied. On the other hand, the temperature effects on the band gap also need to be corrected, as most experiments are performed at room temperature while DFT applies to *T* = 0 *K*. According to the experimental work of Martin *et al*.^30^, the band gap of SnSe changes almost linearly with temperature. By extrapolating the experimental data to *T* = 0 *K*, we obtain a band gap of 0.992 *eV*. Therefore, the theoretical result obtained using GGA combined with the mBJ method is in best agreement with the experimental value. Using GGA-mBJ, we have also calculated the band gap of the *Cmcm* phase at ambient pressure; a smaller band gap of 0.576 *eV* is found, in agreement with recent calculations^26^,^31^.

**Figure 7:**
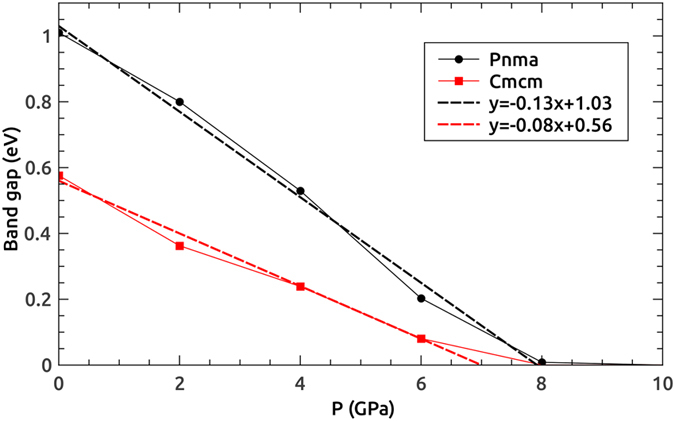
Variation of band gaps as functions of hydrostatic pressure for the *Pnma* and *Cmcm* phases of SnSe. The results are obtained using the GGA-mBJ method. Dashed lines are linear fittings to the DFT data.

Figure 7 shows the theoretical band gaps of the *Pnma* and *Cmcm* phases calculated using GGA-mBJ as a function of hydrostatic pressure. It is seen that the band gaps of both phases decrease linearly with increasing external pressure, while *Pnma* is more sensitive to pressure comparing to *Cmcm*. Eventually, SnSe becomes a zero band gap metal at approximately 8 *GPa*. The linear relationships between the band gaps and hydrostatic pressure are also in consistence with previous experiments^30^. Furthermore, a smaller band gap of the *Cmcm* phase indicates better electrical conductivity comparing to the *Pnma* phase, which may result in a larger power factor. The influences of the varying band gaps on Seebeck coefficients are thus further discussed using the Boltzmann transport theory in the following section.

### Electrical transport properties

As the low temperature phase (*Pnma*) of SnSe transforms to the *Cmcm* phase between 750 *K* ~ 800 *K*^4^,^10^, we focus on different crystal structures when we calculate the electrical transport properties in different temperature ranges. In particular, we consider the *Pnma* structure in the temperature range of 200 *K* ~ 750 *K*, and the *Cmcm* structure in the temperature range of 750 *K* ~ 1000 *K*. As discussed in previous sections, the phase transition temperature decreases as external hydrostatic pressure is applied. Based on AIMD simulations, the transformation from *Pnma* to *Cmcm* completes at 500 *K* under a hydrostatic pressure of 4 *GPa*.

**Figure 8:**
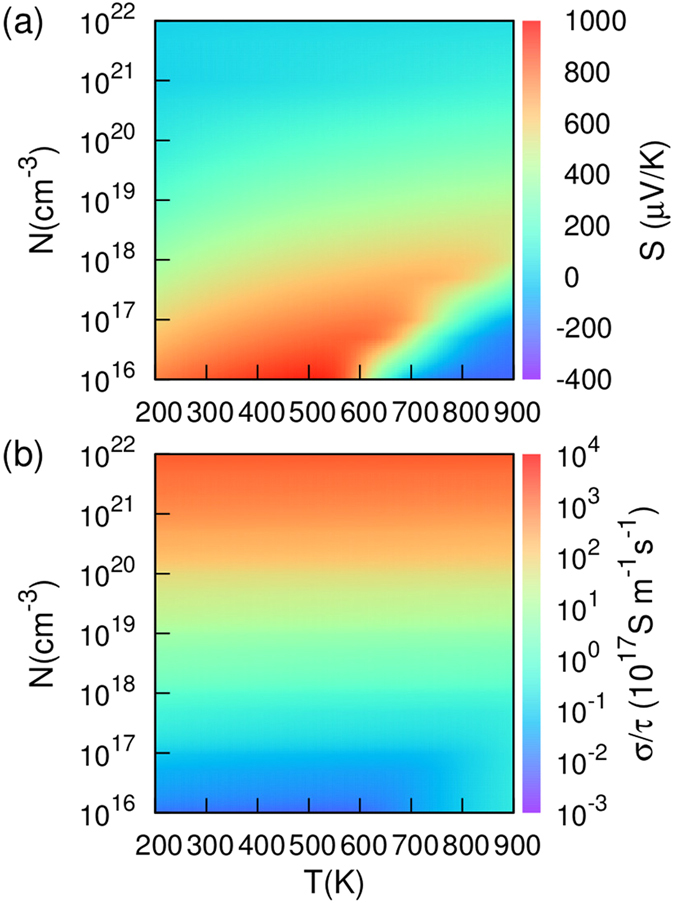
Seebeck coefficient (**a**) and electrical conductivity (**b**) of the *Pnma* phase of SnSe as functions of temperature and carrier concentration.

**Figure 9:**
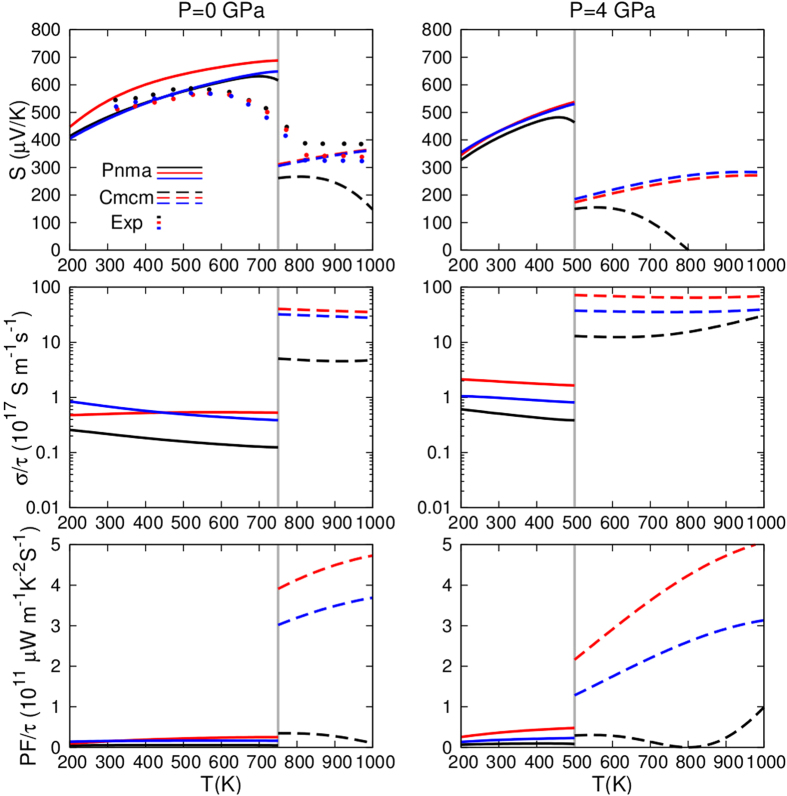
Seebeck coefficient (upper), electrical conductivity (middle) and power factor (bottom) of SnSe under different pressures. Solid and dashed lines represent the *Pnma* and *Cmcm* phases, respectively. Black, red and blue lines represent the orientation of *a, b* and *c* axes. Discrete symbols are experimental data taken from ref. 4.

It is known that SnSe single crystal is a p-type semiconductor, and the main charge carriers are holes. The Seebeck coefficient and electrical conductivity divided by relaxation time of the *Pnma* phase at 0 *GPa* are calculated using the Boltzmann transport theory, as shown in Fig. 8. It is seen that both quantities depend strongly on carrier concentrations, while the Seebeck coefficient is more sensitive to temperature comparing to the electrical conductivity. As expected, the Seebeck coefficient decreases as hole concentration is increased, while the electrical conductivity exhibits an opposite trend. In general, carrier concentration varies as temperature changes. In this study, we have chosen a fixed carrier concentration of *N* = 6*E*17 *cm*^−3^ for the *Pnma* phase and *N* = 2*E*19 *cm*^−3^ for the *Cmcm* phase; these values are determined based on the inverse Hall coefficient measurements^4^. Using these fixed carrier concentrations, we have calculated the Seebeck coefficients of SnSe as a function of temperature, as shown in Fig. 9. A good agreement with the experimental Seebeck coefficient values is obtained; the relatively large discrepancy near the phase transition temperature is due to the fact that the structural phase transition is a continuous process and intermediate crystal structures exist in the middle temperature range.

Electrical conductivity and power factor divided by relaxation time have also been calculated. It is seen from Fig. 9 that the electrical conductivities of both *Pnma* and *Cmcm* are relatively insensitive to temperature, while the *Cmcm* phase has a much larger electrical conductivity. In other words, the experimentally observed increase in electrical conductivity between 700 *K* ~ 800 *K* is mainly due to the structural phase transition. A smaller band gap of the *Cmcm* phase is responsible for its higher electrical conductivity. Along the inner-layer directions (*b* and *c* axes), the electrical conductivity has similar values, while in the inter-layer direction (*a* axis), the electrical conductivity is much smaller; these comparisons are consistent with experiments^4^.

The influences of hydrostatic pressure on the electrical transport properties of SnSe are also investigated. The Seebeck coefficients, electrical conductivities and power factors of both *Pnma* and *Cmcm* phases are calculated after the system is relaxed under a hydrostatic pressure of 4 *GPa*; the results are shown together with those for the ambient pressure in Fig. 9. It is seen that the Seebeck coefficients of the *Pnma* phase in the low temperature range decreases slightly as the hydrostatic pressure is applied. In the high temperature range, where the *Cmcm* phase becomes stable, the relevant Seebeck coefficients do not have obvious changes regarding the external pressure, except the values along the *a* axis. Nonetheless, the transport properties along the *a* axis is of less interest, as the corresponding thermal energy conversion efficiency is relatively low^4^. Hydrostatic pressure in general increases the electrical conductivities of both *Pnma* and *Cmcm*, as a result of the induced smaller band gaps.

It is found that the power factor of the *Pnma* phase is much smaller than that of the *Cmcm* phase. The power factor is strongly anisotropic, and it has much larger values along the inner-layer directions comparing to the inter-layer direction. The overall effects of a 4 *GPa* hydrostatic pressure on the power factor is rather small, however, the phase transition temperature from *Pnma* to *Cmcm* is significantly lower. In other words, applying a small hydrostatic pressure on SnSe stabilizes the *Cmcm* phase at a relatively lower temperature, resulting in a dramatic increase in the power factor in the middle temperature range.

### Thermal transport properties

The ultra-low thermal conductivity of SnSe also contributes to its unprecedentedly high *ZT* value. It is thus of interest to investigate the effects of hydrostatic pressure on the thermal transport, which is dominated by the lattice component (see Fig. 2d in ref. 4). It is known that the lattice thermal conductivity is closely related to the anharmonic potential which determines the phonon-phonon interactions^4^,^32^,^33^. Without specifically calculating all interacting terms, *Grüneisen* parameters are usually good indicators for the lattice anharmonicity.

**Figure 10:**
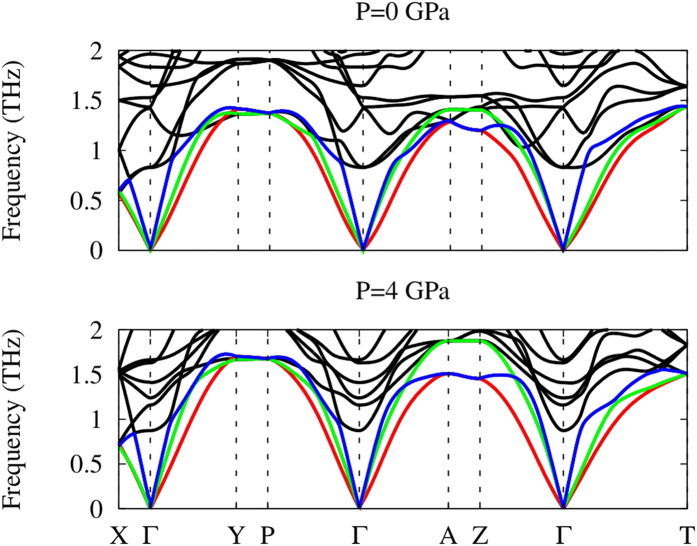
Phonon dispersions of the *Pnma* phase at different pressures. The red, green and blue lines represent TA, TA’ acoustic phonon branches and LA longitudinal acoustic phonon branch, respectively.

**Figure 11:**
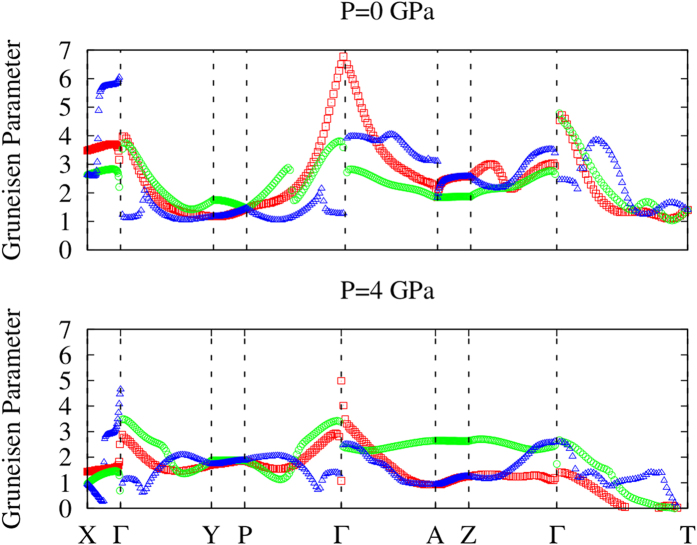
*Grüneisen* dispersions of the *Pnma* phase at different pressures. The red, green and blue symbols represent TA, TA’ and LA modes, respectively.

The phonon and *Grüneisen* dispersions under different pressures are compared in Figs 10 and 11. Due to the dynamical instability of the *Cmcm* phase at *T* = 0 *K*, we have only considered the *Pnma* phase. It is seen from the phonon dispersions that the acoustic modes along the *a* axis (Γ − *X*) are softer than those along the *b* axis (Γ − *Y*) and *c* axis (Γ − *Z*), which confirms that the chemical bonding along the inter-layer direction (*a* axis) is weaker than the inner-layer directions. All of the three acoustic phonon modes become harder when a hydrostatic pressure of 4 *GPa* is applied, indicating stronger chemical bonding under compression. At 0 *GPa*, the average *Grüneisen* parameters are 3.75, 1.9 and 2.6 along the *a, b* and *c* axes, respectively. The large values of the *Grüneisen* parameters suggest strong lattice anharmonicity, rationalizing the intrinsically ultra-low lattice thermal conductivity. The largest *Grüneisen* parameter found along the *a* axis is consistent with the lowest lattice thermal conductivity along this direction. Under a 4 *GPa* hydrostatic pressure, the average *Grüneisen* parameters become 1.70, 1.94 and 1.86 along the *a, b* and *c* axes, respectively. It is interesting to see a larger decrease in the *Grüneisen* parameter along the inter-layer direction, which may be related to the higher compressibility in this direction.

**Figure 12:**
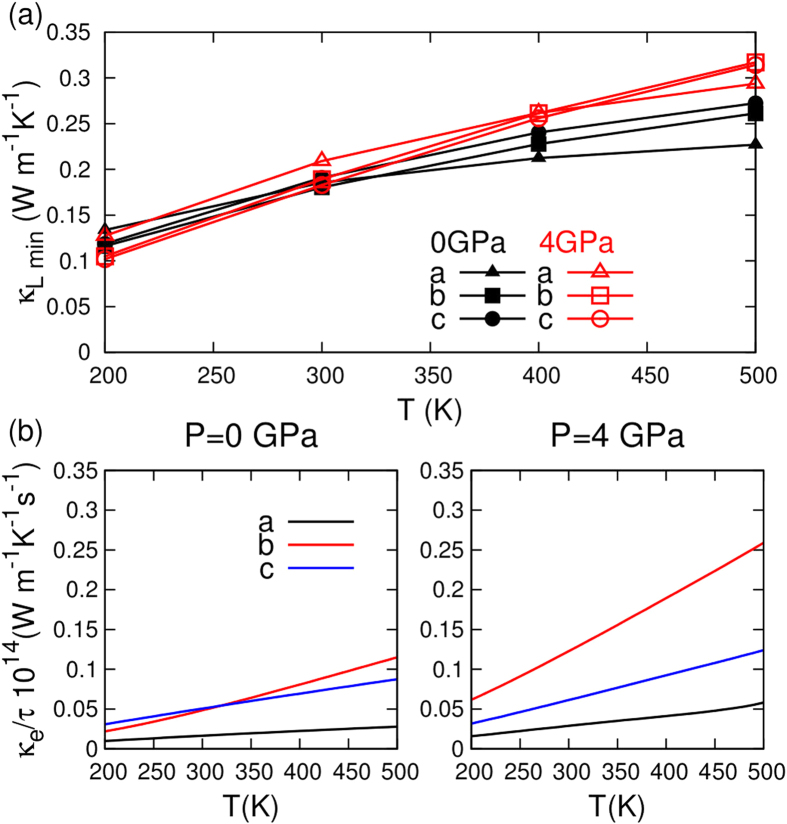
(**a**) Theoretical minimum lattice thermal conductivities of *Pnma* at different pressures. (**b**) Electronic thermal conductivities of the *Pnma* phase as functions of temperature at different pressures.

In general, an increase in group velocity and decrease in *Grüneisen* parameters are found in SnSe when a hydrostatic pressure is applied, therefore, it is anticipated that the lattice thermal conductivity increases under hydrostatic pressure. To further evaluate the pressure effects, we have also calculated the amorphous limit to the thermal conductivity along different axes, as shown in Fig. 12(a). Our calculations at 0 *GPa* are in good agreement with previous publication^4^. More importantly, we show that the amorphous limits of the thermal conductivity only have small increases under pressure. In other words, our results imply that a hydrostatic pressure of 4 *GPa* do not significantly increase the lattice thermal conductivity of SnSe, which is beneficial for the enhancement of the *ZT* value.

Besides lattice thermal conductivity, we have also investigated the effects of hydrostatic pressure on the electronic component based on the Boltzmann transport theory (Fig. 12(b)). It is found that the *Pnma* phase has an extremely small electronic thermal conductivity and the anisotropy is small at 0 *GPa* but becomes more apparent at 4 *GPa. κ*_*e*_/*τ* along *b* and *c* are greater than the one along *a*, in consistence with experiments^4^. Although the electronic thermal conductivity has a small increase under pressure, the absolute value is very small, and it does not affect the overall thermal conductivity significantly.

## Conclusion

The effects of pressure on the structural phase transition and transport properties of SnSe have been investigated based on DFT, AIMD and Boltzmann transport theory. It is found that hydrostatic pressure can effectively reduce the transformation temperature from the *Pnma* to the *Cmcm* phase, leading to an significant enhancement in the power factor of SnSe in the middle-temperature range. We have also found that although uniaxial stress reduces the energy difference between the two competing structures, the *Cmcm* phase is dynamically unstable. The band gap of SnSe has been accurately reproduced using the mBJ exchange potential. Based on Boltzmann transport theory, we successfully reproduce the experimental Seebeck coefficients and the strongly anisotropic electrical transport properties of SnSe. By further calculating the amorphous limit and the electronic component of thermal conductivity, we find that the transport properties of SnSe are relatively unsensitive to hydrostatic pressure. Comparing to the *Pnma* phase, *Cmcm* has a smaller Seebeck coefficient but a much greater electrical conductivity, which results in a higher power factor in the *Cmcm* phase. It is predicted that the thermoelectric efficiency of SnSe in the middle-temperature range can be further enhanced by applying a small hydrostatic pressure, providing an alternative method for achieving higher device *ZT* value.

## Additional Information

**How to cite this article**: Yu, H. *et al*. Enhanced power factor via the control of structural phase transition in SnSe. *Sci. Rep.* **6**, 26193; doi: 10.1038/srep26193 (2016).

## Supplementary Material

Supplementary Information

## Figures and Tables

**Table 1:** Electronic band gaps of the *Pnma* phase of SnSe calculated using different methods.

	GGA	GGA + mBJ	LDA	LDA + mBJ	Expt.
*E*_*gap*_(*eV*)	0.602	1.006	0.326	0.654	0.992

The extrapolated experimental value at T = 0 *K* is also shown for comparison.
