# Supplementary material for: Enhanced power factor via the control of structural phase transition in SnSe
Source: Sci Rep. 2016 May 19;6:26193. doi: 10.1038/srep26193 (PMC4872138; doi:10.1038/srep26193)
Supplement: Supplementary Information [file srep26193-s1.pdf]

Supplemental Information
Enhanced power factor via the control of structural phase transition in SnSe

Hulei YU[1], **Shuai DAI**[1], **and Yue CHEN**[1,*]

[1]Department of Mechanical Engineering, The University of Hong Kong, Pokfulam Road, Hong Kong SAR, China
[*]yuechen@hku.hk

Structural phase transition under hydrostatic pressure

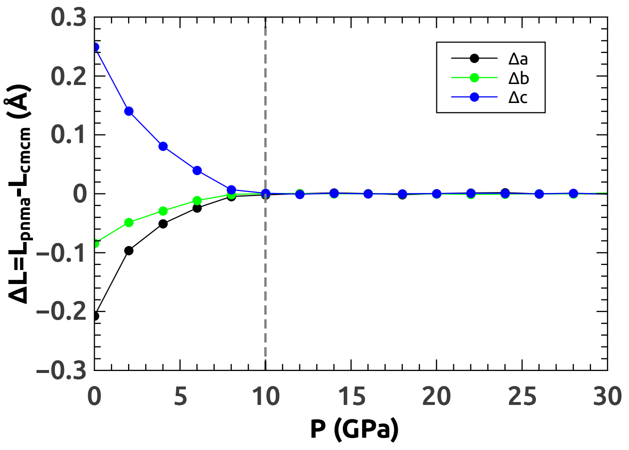

Figure S1. Lattice parameters (L) as a function of hydrostatic pressure. The lattice parameters of the *Cmcm* phase are taken as references.

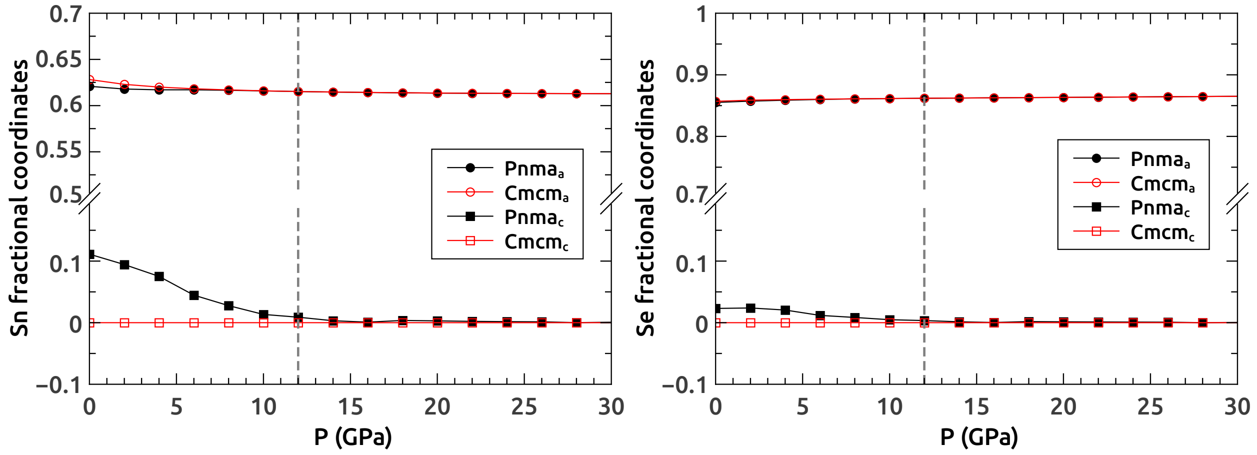

Figure S2. Fractional coordinates of the Sn (left) and Se (right) atoms as a function of hydrostatic pressure.

The lattice parameters (Figure S1) and atomic positions (Figure S2) of the *Pnma* and *Cmcm* phases are compared under different hydrostatic pressures to provide direct evidences of the structural phase transition. Due to symmetry constraints, Sn and Se have the same fractional coordinates along the b axis in both of the *Pnma* and *Cmcm* structures; therefore, we have only shown the atomic coordinates along the a and c axes in Figure S2. Obvious differences in the lattice parameters and atomic coordinates exist at ambient pressure, while the *Pnma* phase gradually transforms to the *Cmcm* phase under hydrostatic pressure. The phase transition is found to complete at a pressure around $12GPa$.

1 The effects of uniaxial stress

Uniaxial stress has been applied along three different axes of the unit cells in both *Pnma* and *Cmcm* phases. It is found that the phase transition from *Pnma* to *Cmcm* can be induced only when the applied uniaxial stress is along the c direction. The energy differences between the two competing structures are shown in Figure S3. It is seen that *Pnma* is initially energetically preferable, while its energy becomes identical to that of *Cmcm* at approximately $3.5GPa$. The changes in lattice parameters and atomic coordinates are shown in Figure S4 and Figure S5. It is seen that *Pnma* transforms to *Cmcm* at a pressure around $3.5GPa$. On the other hand, as discussed in the paper, the *Cmcm* phase is dynamically unstable under uniaxial stress.

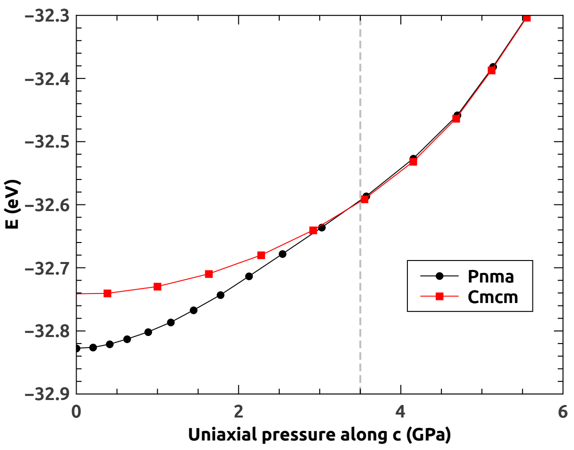

Figure S3. Energy differences between the *Pnma* and *Cmcm* phases under uniaxial stress along the *c* axis.

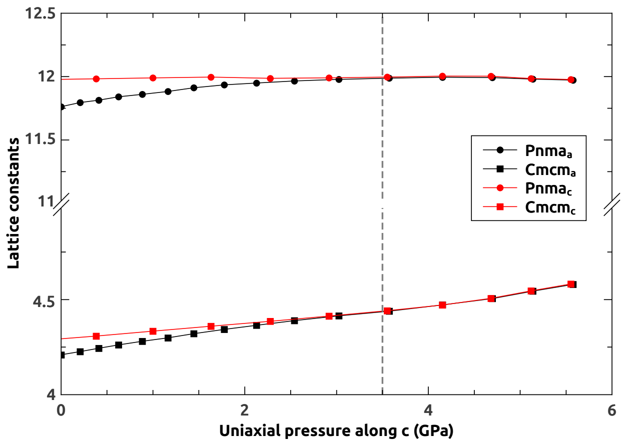

Figure S4. Lattice constants of the *Pnma* and *Cmcm* phases as functions of uniaxial stress along the *c* axis.

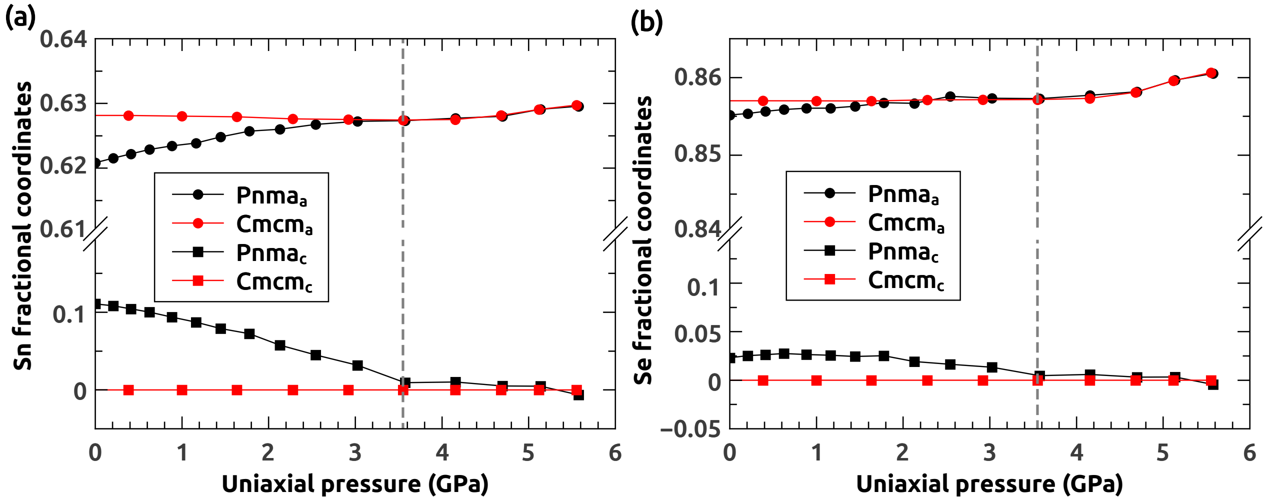

Figure S5. Fractional coordinates of the Sn (a) and Se (b) atoms as functions of uniaxial stress along the *c* axis.

2 Transport properties

The effects of carrier concentration on the Seebeck coefficients of the *Pnma* phase is shown in Figure S6. The anisotropic behavior in the electrical transport is clearly seen. As expected, the Seebeck coefficients decrease with increasing carrier concentration. In general, Seebeck coefficients are relatively unsensitive to temperature before a sudden drop observed at low carrier concentrations. Debye temperature, group velocity as well as Grüneisen parameter are summarized in Table S1. Debye temperature is calculated with $\theta = \omega_D/k_B$, where ω_D is the largest acoustic phonon frequency in different directions, and k_B is the Boltzmann constant.

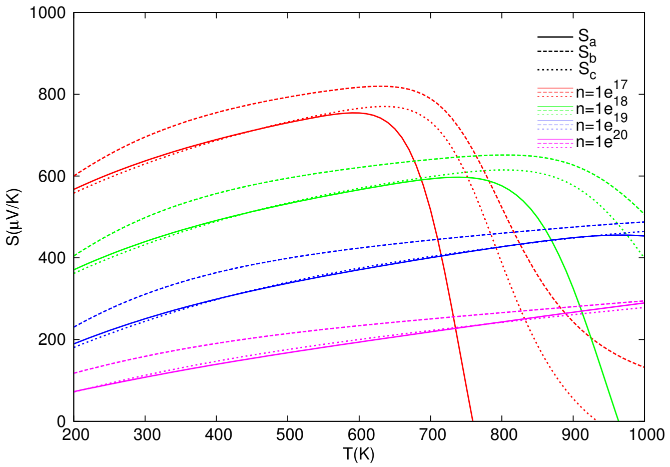

Figure S6. Seebeck coefficients of the *Pnma* phase with different carrier concentrations.

$\Gamma X/a$	θ (K) 0GPa	4GPa	v (m/s) 0GPa	4GPa	γ 0GPa	4GPa
TA	27.3	35	1453	1896	2.74	1.56
TA'	28.6	33.6	1575	1997	3.61	1.29
LA	33.8	40.3	1252	1804	4.91	2.24
Average	29.9	36.3	1427	1899	3.75	1.7
$\Gamma Y/b$						
TA	68	81.7	1149	1621	2.1	1.85
TA'	66	80.1	1687	2605	2.31	2.35
LA	68.5	83	3175	3601	1.26	1.63
Average	67.5	84.6	2003	2609	1.9	1.94
$\Gamma Z/c$						
TA	57.6	69.8	1223	1438	2.7	1.27
TA'	67.5	90.1	2289	3180	2.33	1.82
LA	60.1	71.8	2358	3388	2.78	2.5
Average	61.7	77.2	1957	2669	2.6	1.86

Table S1. Transverse (TA/TA') and longitudinal (LA) Debye temperatures (θ), group velocities (v) and Grüneisen parameters (γ) along the *a*, *b* and *c* axes of the *Pnma* phase
